# Identification of four novel small non-coding RNAs from *Xanthomonas campestris *pathovar *campestris*

**DOI:** 10.1186/1471-2164-11-316

**Published:** 2010-05-20

**Authors:** Rui-Ping Jiang, Dong-Jie Tang, Xiao-Lin Chen, Yong-Qiang He, Jia-Xun Feng, Bo-Le Jiang, Guang-Tao Lu, Min Lin, Ji-Liang Tang

**Affiliations:** 1 Guangxi Key Laboratory of Subtropical Bioresources Conservation and Utilization, Guangxi University, 100 Daxue Road, Nanning, Guangxi 530004, China; 2 The Key Laboratory of Ministry of Education for Microbial and Plant Genetic Engineering, Guangxi University, 100 Daxue Road, Nanning, Guangxi 530004, China; 3 College of Life Science and Technology, Guangxi University, 100 Daxue Road, Nanning Guangxi 530004, China; 4 Biotechnology Research Institute, Chinese Academy of Agricultural Sciences, Beijing, 100081, China

## Abstract

**Background:**

In bacteria, small non-coding RNAs (sRNAs) have been recognized as important regulators of various cellular processes. Approximately 200 bacterial sRNAs in total have been reported. However, very few sRNAs have been identified from phytopathogenic bacteria.

**Results:**

*Xanthomons campestris *pathovar *campestris *(*Xcc*) is the causal agent of black rot disease of cruciferous crops. In this study, a cDNA library was constructed from the low-molecular weight RNA isolated from the *Xcc *strain 8004 grown to exponential phase in the minimal medium XVM2. Seven sRNA candidates were obtained by sequencing screen of 2,500 clones from the library and four of them were confirmed to be sRNAs by Northern hybridization, which were named sRNA-*Xcc*1, sRNA-*Xcc*2, sRNA-*Xcc*3, and sRNA-*Xcc*4. The transcription start and stop sites of these sRNAs were further determined. BLAST analysis revealed that the four sRNAs are novel. Bioinformatics prediction showed that a large number of genes with various known or unknown functions in *Xcc *8004 are potential targets of sRNA-*Xcc*1, sRNA-*Xcc*3 and sRNA-*Xcc*4. In contrast, only a few genes were predicted to be potential targets of sRNA-*Xcc*2.

**Conclusion:**

We have identified four novel sRNAs from *Xcc *by a large-scale screen. Bioinformatics analysis suggests that they may perform various functions. This work provides the first step toward understanding the role of sRNAs in the molecular mechanisms of *Xanthomonas campestris *pathogenesis.

## Background

Numerous evidences show that small non-coding RNAs (sRNAs) exist in all three domains of life, i.e. Eukarya, Bacteria and Archaea. Bacterial sRNAs are normally between 50 and 500 nucleotides in length. It has been demonstrated that many bacterial sRNAs act as regulators of gene expression, although the function of the majority of identified bacterial sRNAs is still unknown. Recent studies have revealed that in bacteria sRNAs control various cellular processes, including acid resistance [1], iron homeostasis[2], sugar metabolism[3], envelope stress response [4,5], quorum sensing [6], as well as virulence [7,8]. Most bacterial sRNAs characterized to date regulate gene expression either by pairing to their mRNA targets and thus affecting their stability and/or translation, or by binding to proteins to modify their mRNA-binding activity [9,10].

Several experimental strategies have been employed to identify sRNAs[11,12] and approximately two hundred bacterial sRNAs in total have been discovered thus far. Among the identified bacterial sRNAs, almost half were from *Escherichia coli *[12,13], and the other half were mainly from *Bacillus subtilis *[14], *Caulobacter crescentus *[15], *Listeria monocytogenes *[16], *Mycobacterium tuberculosis *[17], *Pseudomonas aeruginosa *[18], *Salmonella typhimurium *[19], *Sinorhizobium meliloti *[20,21], *Staphylococcus aureus *[22], and *Vibrio cholerae *[23]. Based on bioinformatics analysis, it is estimated that per bacterial genome may encode several hundred sRNAs[24,25]. Thus, there are still a large number of unknown sRNAs in bacteria including the standard model bacterium *E. coli*. Very few sRNAs have been reported from plant pathogenic bacteria.

The Gram-negative bacterium *Xanthomonas campestris *pathovar *campestris *(*Xcc*) is the causal agent of black rot disease of cruciferous crops worldwide[26]. This pathogen infects almost all the members of crucifer family (*Brassicaceae*), including important vegetables such as broccoli, cabbage, cauliflower, mustard, and radish; the major oil crop rape; and the model plant *Arabidopsis thaliana*. In recent decades, the black rot disease has become more prevalent and caused severe losses in vegetable and edible oil productions in many countries[27]. In addition, *Xcc *is the producer of the acid exopolysaccharide xanthan, which is an important industrial biopolymer and has been widely used as a viscosifer, thickener, emulsifier or stabilizer in both food and non-food industries[28]. Because of its agricultural and industrial importance, molecular genetics of *Xcc *has attracted particular attention for over two decades. The entire genome sequences of three *Xcc *strains have been determined and many important genes implicated in pathogenicity, xanthan biosynthesis, and other cellular processes have been characterized [27,29-33]. However, no sRNA has been identified from *Xcc *so far. In this article, we report four sRNAs identified from the *Xcc *strain 8004 by generating and screening a cDNA library of low molecular weight RNAs, providing the first step towards an understanding of the function of sRNAs in *Xanthomonas*.

## Results and discussion

### Construction of a cDNA library of low molecular weight RNAs from *Xcc*

As mentioned above, to identify sRNAs in *Xcc *we employed the approach based on a cDNA library of low molecular weight RNAs (Additional file 1 Figure S1). This strategy, also known as small RNA shotgun cloning, allows detection of sRNAs that are expressed in the bacterial cells grown at given conditions but does not require prior knowledge of sRNA characteristics [12,25]. This method has been proven to be one of the most efficient ways for sRNA identification in bacteria[12,25]. We constructed a cDNA library by reverse-transcribing the RNAs with size ranging from about 50 to 500 nt, which were selected from the total RNA isolated from the bacterial cells of the *Xcc *strain 8004 [34] grown to the exponential phase in the medium XVM2, a minimal medium mimicking plant cells [35]. Since the RNAs with size ranging from 50 to 500 nt overlap in length with the very highly abundant 5S rRNA transcripts (119 nt), we excised the RNA band with the size about 110 nt from the gel after electrophoresis to deplete 5S rRNA and enrich for other sRNAs. By using the method described in Methods, a cDNA library containing approximately 10,000 individual clones was constructed.

### Identification of sRNA candidates from the cDNA library

About 2,500 individual clones from the cDNA library were exposed to sequence determination; of which, 2,104 recombinant plasmids with satisfactory cloned sequences were obtained (Additional file 2 Table S1). The obtained insert sequences of these recombinant plasmids were individually aligned by BLASTN against the genomic sequence of *Xcc *strain 8004 on NCBI GenBank database[30] (GenBank accession number CP000050) and 2,048 of them match to the genome (Additional file 2 Table S1). Of the 2,048 matched sequences, 1,274(60.55%) were derived from tRNA genes, 444 (21.1%) from 5S rRNA genes, 67 (3.18%) from the 16S or 23S rRNA genes, 6 (0.29%) from ORF (open reading frame)-coding regions, and 257 (12.22%) from intergenic regions (IGRs) (Table 1 and Additional file 2 Table S1 to Table S4). The sequences of the 6 ORF-matched clones all correspond to the sense orientation; therefore, it is probable that they are degeneration products of the full length mRNAs encoded by the ORFs. The 257 IGR-matched clones are comprised 7 species. We considered these species as potential candidates of sRNAs and named them sRNA-C1 to sRNA-C7, respectively (Table 2 and Additional file 2 Table S4).

**Table 1:** Distribution of the cDNA clones on the genome of Xcc strain 8004

Category	Clone number	Kinds	% of total clones
5S rRNA	444	1	21.10
16S/23S rRNA	67	2	3.18
tRNAs	1274	43(53)^a^	60.55
Intergenic region	257	7	12.22
Protein-coding genes or ORFs	6	6	0.29
Unmatched	56		2.66
Total	2104		100

^a^A total of 53 tRNAs were annotated in the genome of *Xcc *8004[30].

**Table 2:** A summary of the analysis of the sRNA candidate

sRNA candidate	Clone number	Intergenic region	strand	Clone(s) size (nt)^a^	Northern size (nt)^b^	sRNA?	5' end^c^	3' end^c^	sRNA size (nt)^d^
sRNA-C1	1	XC0350-XC0351	+	83	~100	Yes (sRNA-*Xcc*1)	410075	410162	88
sRNA-C2	214	XC0901-XC0902	-	187	~200	Yes (sRNA-*Xcc*2)	1085474	1085288	187
sRNA-C3	2	XC3244-XC3245	-	70	~100, >1000	Yes (sRNA-*Xcc*3)	3890134	3890025	110
sRNA-C4	5	XC3924-XC3925	-	36	~100, >1000	Yes (sRNA-*Xcc*4)	4632459	4632338	122
sRNA-C5	3	XC4108-XC4109	-	20	>1000	No	ND	ND	
sRNA-C6	28	XC4382-XC4383	-	70	>1000	No	ND	ND	
sRNA-C7	4	XC4385-XC4386	-	63	>1000	No	ND	ND	

^a^The size of the largest clone if more than 1 clone obtained.

^b^Size observed on Northern blots.

^c^Ends determined by 5'- or 3'- RACE mapping.

^d^Size calculated according to the 5'- and 3'-ends determined by RACE mapping.

ND, not done.

It is not surprising that 60.55% of the clones were derived from tRNA genes, because the RNAs used for the cDNA library construction overlap in length with the highly abundant tRNA transcripts (about 70 nt in size), which were not removed from the RNA templates used for reverse-transcription in the library construction. The genome of the *Xcc *strain 8004 harbours 53 copies of tRNA genes consisting of 46 species distinguishable in sequences[30]. The sequences of the 1,274 clones derived from tRNA genes match respectively to 43 different tRNA species (Table 1 and Additional file 2 Table S2). On the contrary, it is surprising that there are still 21.1% of the clones are 5S rRNA transcripts, although the 5S rRNA-included band was removed from the RNA fractionization gel during the cDNA library construction.

### Identification of sRNAs from the candidates by Northern blotting

**Figure 1:**
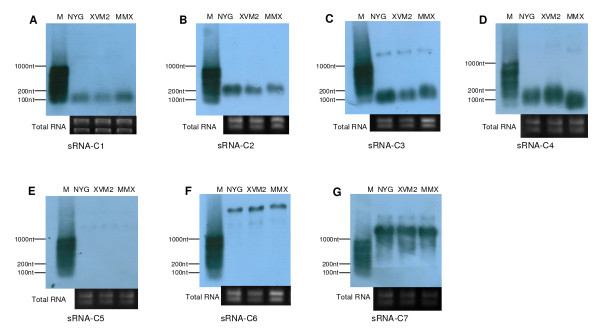
**Northern blots verify the presence of *Xcc *sRNAs**. RNA samples isolated from exponential phase cells cultured in NYG, XVM2 and MMX medium were analyzed by Northern blotting using DNA probes complementary to cDNA clones of the sRNA candidates (for more detail see Methods). A to G represent the Northern blotting results using DNA probes complementary to cDNA clones of the sRNA candidates sRNA-C1 to sRNA-C7, respectively. Transcript sizes are approximate and compared to RiboRulerTM RNA ladder Low Range (Fermentas) that labeled by Turbo LabelingTM Kit (KPL) (M). Corresponding ethidium bromide stained gels show equal loading of total RNA in all lanes.

**Figure 2:**
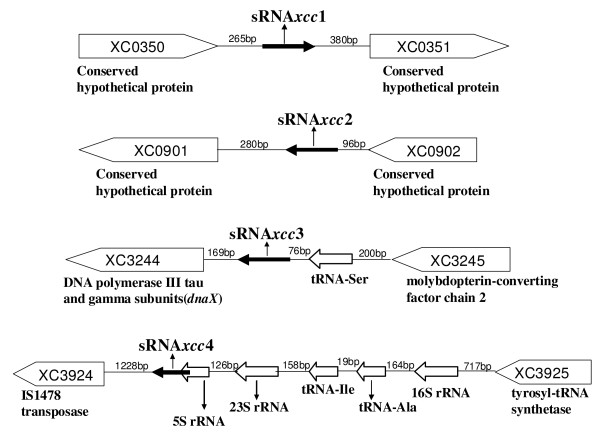
**Genomic positions of the identified sRNA genes**. Schematic showing genome locations of the four sRNAs in the *Xcc *strain 8004. sRNA genes are shown as black arrows with names of the sRNAs under the arrows. The distances between a sRNA gene and its up and downstream genes and the genome ID of the flanking genes are indicated (the annotated function of the flanking genes are shown under the arrow). The orientation of the arrow indicates the transcriptional orientation of the corresponding gene.

To further verify if the 7 sRNA candidates identified from the cDNA library were authentic sRNAs, we performed Northern blotting analysis using DNA probes complementary to the original cDNA clones of the candidates. The results showed that a single Northern blotting signal band was clearly observed for each of the sRNA candidates, sRNA-C1 and sRNA-C2, and the sizes of the bands were approximately 100 and 200 nt in length, respectively (Figure 1), which are consistent with the sizes of the corresponding candidate cDNAs (Table 2). We concluded that these two candidates are genuine sRNAs and named them sRNA-*Xcc*1 and sRNA-*Xcc*2, respectively (Table 2). For each of the candidates, sRNA-C3 and sRNA-C4, two Northern blotting signal bands were observed; a major band with small size and a very faint band with large size (Figure 1). As shown in Figure 1, the sizes of the major bands were about 50 and 100 nt in length, respectively, which are consistent with the sizes of the corresponding candidate cDNAs (Table 2). We concluded that they are real sRNAs and named them sRNA-*Xcc*3 and sRNA-*Xcc*4, respectively. The faint bands might result from artificial hybridization of the sRNA-C3 and sRNA-C4 probes with unknown transcripts. The blots of the candidates sRNA-C5, sRNA-C6 and sRNA-C7 showed signal band(s) larger than 1000 nt, much larger than the sizes of the cDNAs, thus they are not like to be sRNAs. A summary of the analysis of these sRNA candidates is presented in Table 2, and the locations of the four verified sRNAs in the genome of the *Xcc *strain 8004 are shown in Figure 2. Interestingly, the gene encoding sRNA-*Xcc*4 overlaps with the 5S rRNA gene.

To gain a clue to understanding the expression of the identified sRNAs, we compared the expression levels of the sRNAs in the bacterial cells grown to exponential phase in different media by Northern blotting analysis. As shown in Figure 1, the expression levels of the four sRNAs in the rich medium NYG and the minimal media MMX and XVM2 are very high and almost identical. This suggests that in exponential growth phase the expression of the four *Xcc *sRNAs is not nutrition dependent.

### 5' and 3' end mapping, secondary structure prediction, and target prediction of the identified sRNAs

Northern blots only provide information about the expression level and the approximate size of a transcript, but can not detect the exact position of the 5' and 3' ends of RNA. To precisely ascertain the transcription start and stop sites of the identified sRNAs, 5' and 3' RACE analysis was performed (see Methods for details). The results are given in Additional file 3 Tables S5 and S6. Since 5' and 3' ends of a sRNA may vary by a few nucleotides, at least 10 clones for each 5' and 3' RACE analysis should be sequenced, and the most upstream 5' nucleotide is regarded as the transcription initiation site and the most downstream 3' nucleotide is regarded as the transcription termination site. The 5' and 3' termini of the four identified sRNAs were determined by the above strategy and shown in Table 2.

**Figure 3:**
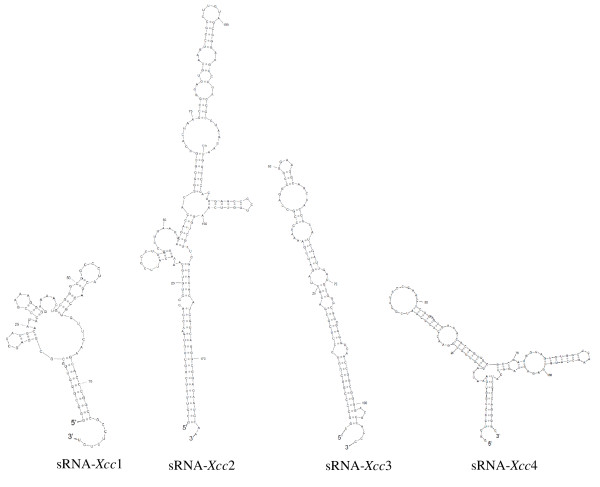
**The predicted secondary structures of Xcc sRNAs**. The secondary structures of the four identified *Xcc *sRNAs were predicted by using SFold program [36].

After the 5' and 3' termini of the transcripts were identified, we assigned the most probable boundaries for the sRNAs, and the secondary structure of each of the resulting sequences was analyzed by using SFold [36]. The predicted secondary structures of the four *Xcc *sRNAs are shown in Figure 3.

It has been demonstrated that most characterized sRNAs regulate gene expression by pairing to their mRNA targets[9,10]. As a first step in gaining an understanding of the function of the identified *Xcc *sRNAs, we employed the computational software sRNATarget developed by Cao and associates[37] to predict their potential targets. The results, as shown in Additional file 4 Table S7, reveal that a large number of genes with various predicted or known functions, including some known virulence-related genes, are potential targets of sRNA-*Xcc*1, sRNA-*Xcc*3 and sRNA-*Xcc*4, suggesting that these sRNAs are probably implicated in the regulation of different cellular processes including pathogenesis. In contrast, only a few genes were predicted (at a very low score) to be potential targets of sRNA-*Xcc*2, implying that sRNA-*Xcc*2 might be a structural rather than a regulatory RNA. To ascertain the indisputable biological significance of these sRNAs in *Xcc *needs further experimental investigations.

### Distribution of the identified sRNA genes in other bacteria

To determine whether the sRNAs identified above have any sequence similarity to other known bacterial sRNAs, a BLAST[38] was used to search the sequences of the sRNAs against the small RNA database http://ncrnadb.trna.ibch.poznan.pl/blast.html. None of the four identified sRNAs displayed sequence similarity with any known sRNAs, indicating that these four *Xcc *sRNAs are novel. To further verify whether homologous DNA sequences of these sRNA coding genes exist in other microorganisms, we used the complementary DNA sequences of these sRNA genes to perform BLAST searches against the NCBI total sequence database http://www.ncbi.nlm.nih.gov/Genbank/index.html. The result, which is given in Table 3, showed that: (i) to sRNA-*Xcc*1, homologous sequences were only found in the genomes of the *Xcc *strains ATCC33913 and B100 but not in any other sequenced bacterial species including the very closely related bacteria *X. campestris *pv. *vesicatoria *and *X. oryzae *pv. *oryzae*, indicating that sRNA-*Xcc*1 may be an *Xcc *specific sRNA; and (ii) to sRNA-*Xcc*2, sRNA-*Xcc*3 and sRNA-*Xcc*4, highly homologous sequences were found in other species of *Xanthomonas *and its closely related genus *Xylella*, in addition to the *Xcc *strains ATCC33913 and B100; however, no homologous sequence was found in any other bacteria.

**Table 3:** Distribution and conservation of the identified sRNA gene sequence in closely related bacteria

Bacterial species	***Xcc***		***Xcv***	***Xoo***			***Xf***		***Xac***
Strain	ATCC33913	B100	85-10	KACC10331	MAF311018	PXOO99A	9a5c	Temecule1	306
sRNA-*Xcc*1	100%	87%	N	N	N	N	N	N	N
sRNA-*Xcc*2	100%	98%	95%	94%	94%	94%	84%	84%	94%
sRNA-*Xcc*3	100%	100%	100%	100%	99%	99%	91%	91%	100%
sRNA-*Xcc*4	100%	100%	100%	100%	100%	100%	95%	94%	100%

*Xcc, X. campestris *pv. *campestris*; *Xcv*, *X. campestris *pv. *vesicatoria*; *Xoo*, *X. oryzae *pv. *oryzae*; *Xf*, *Xylella fastidiosa*; *Xac*, *Xanthomonas axonopodis *pv. *citri*, N, no similar sequence found.

## Conclusion

A cDNA library was constructed with the low molecular weight RNA prepared from the *Xcc *cells grown to exponential phase in the minimal medium XVM2 and seven sRNA candidates were obtained by sequencing screen of approximately 2,500 clones randomly selected from the library. Four of the candidates were confirmed to be sRNAs by Northern blotting. Bioinformatics analysis revealed that all of the four sRNAs are novel. Their transcription start and stop sites were further determined by 5'- and 3'-end mapping. The secondary structure and potential targets of the four sRNAs were predicted bioinformaticsly, suggesting that a large number of genes related to various cellular processes of *Xcc *may be regulated by the sRNAs. To the best of our knowledge, this is the first report on identification of sRNAs from a plant pathogen by a large-scale screen. The results provide useful information for further studies on the molecular mechanisms of *Xanthomonas campestris *pathogenesis.

## Methods

### Bacterial strains, plasmids and growth conditions

The *E. coli *strain JM109 [39] was grown in L medium[40] at 37°С. The *Xcc *strain 8004 [34] was grown in the rich medium NYG [34] and the minimal media MMX [41] or XVM2 [35] at 28°С. Antibiotics were used at the following final concentrations: ampicillin, 100 μg/ml; rifampicin, 50 μg/ml.

### cDNA library construction

A cDNA library of the *Xcc *low molecular weight RNA was constructed using the TaKaRa small RNA cloning kit DRR065 (TaKaRa, Dalian, China) and the experimental steps were performed according to the manufacturer's instructions. A schematic diagram displaying the experimental procedure used for the cDNA library construction was shown in Additional file 1 Figure S1. In brief, overnight cultures, grown in the rich medium NYG, of the *Xcc *strain 8004 were diluted to 1/100 in the minimal medium XVM2 and grown at 28°C. Bacterial cells were harvested at OD_600 _= 0.6 (representing exponential phase) and total RNA was isolated using the hot phenol method [42]. 200 μg RNA were subsequently fractionated by denaturing 8% polyacrylamide gel (7 M urea, 0.5 × TBE buffer) electrophoresis (PAGE). The gel containing target RNAs with size ranging from about 50 to 500 nt were excised after removing the RNA band with the size about 110 nt to deplete 5S rRNA, and RNAs were extracted from the excised gel using the small RNA Gel Extraction Kit D9106 (TaKaRa, Dalian, China). The purified RNAs were then dephosphorylated by bacterial alkaline phosphatase (BAP) treatment and a biotin tagged 3' adaptor (5' phosphorylated) (Additional file 5 Table S8) was ligated to the RNA molecules by T4 RNA ligase (Promega, Shanghai, China). The 3' adaptor-containing RNAs were purified using the Strepto Avidin-labelted Magnet Bead MAGNOTEX-SA (TaKaRa, Dalian, China), which binds specifically to biotin, and a 5' adaptor (Additional file 5 Table S8) was ligated to the small RNA by T4 RNA ligase, and again, the 3' and 5' adaptor-containing small RNAs were purified using the Magnet Bead MAGNOTEX-SA. These RNAs were then reverse-transcribed using primer complementary to the 3' linker sequence, and finally PCR amplified using primers on both linkers. The amplified products were gel-extracted and digested using Sse8387 I (TaKaRa, Dalian, China), and cloned into the vector pUC19 [43] and transferred into *E. coli *JM109 by transformation. Transformed bacterial cells were plated on LB plates containing ampicillin and grown overnight. Individual transformants were picked and screened for presence of inserts by colony PCR. Clones with inserts were uses for sequencing analysis.

### DNA sequencing

The cDNA clones were sequenced using the M13 reverse primer and the BigDye terminator cycle sequencing reaction kit (Applied Biosystems, Foster City, CA, USA) on an ABI Prism 377 (Applied Biosystems, Foster City, CA, USA) sequencer.

### Biocomputational analysis

Mapping of the cDNA clones on the genome of the *Xcc *strain 8004 was carried out by performing a BLASTN search against the genome sequence [30] on GenBank database (NCBI GenBank accession number CP000050). The Vector NTI (Invitrogen, Carlsbad, CA, USA) sequence analysis program package was used for sequence alignment. The SFold program[36] was used for RNA secondary structure prediction. sRNA targets were predicted by using the software developed by Cao and associates [37].

### Northern blotting

*Xcc *overnight cultures were diluted 1/100, grown at 28°C in the rich medium NYG and the minimal media XVM2 or MMX, and bacterial cells were harvested at exponential phase (OD_600 _= 1.0 for NYG and 0.6 for MMX or XVM2). Total RNA was isolated using the SV total RNA Isolation System Kit (Promega, Shanghai, China) and treated by DNase. RNA samples (about 30 μg but normalized to equal 5S rRNA hybridization signals in final experiments) were denatured for 10 min at 68°C in RNA sample loading buffer [62.5% (v/v) deionized formamide, 1.14 M formaldehyde, 1.25×MOPS-EDTA-sodium acetate buffer, 200 μg/ml bromophenol blue, 200 μg/ml xylene cyanol FF and 50 μg/ml ethidium bromide] (Sigma, Missouri, USA), separated on agarose (1.5%) gel electrophoresis in 1× Running Buffer (per liter containing: 100 ml 10× MOPS Buffer, 40 ml 37% formaldehyde, and 860 ml DEPC treated H_2_O) (Generay Biotech Co., Ltd, Shanghai, China), and transferred to Biodyne^® ^B nylon Membrane (KPL, Inc., Gaithersburg, MD, USA) by capillary blotting. Membranes were hybridized with gene-specific Biotin-labeled oligodeoxyribonucleotides (Additional file 5 Table S8) using the DetectorTM AP Chemiluminescent Blotting Kit (KPL, Inc., Gaithersburg, MD, USA) according to the manufacturer's instructions, and hybridization signals were visualized by exposure to a medical X-ray film (Super RX, Fujifilm). For each probe, at least three biological repeats of hybridizations were performed.

### 5' and 3' RACE

5'-rapid amplification of cDNA ends (5'RACE) was carried out using the 5'RACE System for Rapid Amplification of cDNA ends kit (Invitrogen), following the manufacturer's instructions. After purified using the Watson Gel Extraction Mini Kit (Watson Biotechnologies, Inc), the PCR products of 5'RACE were cloned into the T-vector pMD18-T (TaKaRa, Dalian, China) and the cloned cDNA fragments were sequenced and analyzed. 3'-RACE was conducted using the TaKaRa Small RNA cloning Kit (TaKaRa, Dalian, China), following the manufacturer's instructions, and the 3'-RACE PCR products were cloned and sequenced using the same method for 5'-RACE. Primers and RNA adaptors used for 5'- and 3'-RACE are listed in Additional file 5 Table S8.

## Authors' contributions

RPJ carried out cDNA library construction, Northern blot analysis, RACE mapping and Data analysis; DJT coordinated the research, participated in library construction, Northern blot analysis, RACE mapping and Data analysis, and wrote the manuscript; JLT conceived the study, coordinated the research, carried out Data analysis and wrote the manuscript; XLC participated in library construction; YQH, JXF, BLJ, GTL and ML contributed to Data analysis. All authors read, approved and made contributions to the manuscript.

## Supplementary Material

Additional file 1 Figure S1. Schematic diagram showing the method used to clone *Xcc *sRNAs. Click here for file

Additional file 2 Table S1 to S4. Sequence analysis of the cDNA library.xls Click here for file

Additional file 3 Table S5 and S6. 5'and 3'- Race sequencing result.xls Click here for file

Additional file 4 Table S7.sRNA target prediction results.xls Click here for file

Additional file 5 Table S8. Primers used in this study.doc Click here for file

## References

[B1] Opdyke JA Kang JG Storz G GadY, a small-RNA regulator of acid response genes in *Escherichia coli* J Bacteriol 2004 186 6698 6705 10.1128/JB.186.20.6698-6705.2004 15466020 PMC522195

[B2] Wilderman PJ Sowa NA FitzGerald DJ FitzGerald PC Gottesman S Ochsner UA Vasil ML Identification of tandem duplicate regulatory small RNAs in *Pseudomonas aeruginosa *involved in iron homeostasis Proc Natl Acad Sci USA 2004 101 9792 9797 10.1073/pnas.0403423101 15210934 PMC470753

[B3] Vanderpool CK Gottesman S Involvement of a novel transcriptional activator and small RNA in post-transcriptional regulation of the glucose phosphoenolpyruvate phosphotransferase system Mol Microbiol 2004 54 1076 1089 10.1111/j.1365-2958.2004.04348.x 15522088

[B4] Vogel J Papenfort K Small non-coding RNAs and the bacterial outer membrane Curr Opin Microbiol 2006 9 605 611 10.1016/j.mib.2006.10.006 17055775

[B5] Valentin-Hansen P Johansen J Rasmussen AA Small RNAs controlling outer membrane porins Curr Opin Microbiol 2007 10 152 155 10.1016/j.mib.2007.03.001 17369078

[B6] Bejerano-Sagie M Xavier KB The role of small RNAs in quorum sensing Curr Opin Microbiol 2007 10 189 98 10.1016/j.mib.2007.03.009 17387037

[B7] Romby P Vandenesch F Wagner EG The role of RNAs in the regulation of virulence-gene expression Curr Opin Microbiol 2006 9 229 236 10.1016/j.mib.2006.02.005 16529986

[B8] Toledo-Arana A Repoila F Cossart P Small noncoding RNAs controlling pathogenesis Curr Opin Microbiol 2007 10 182 188 10.1016/j.mib.2007.03.004 17383223

[B9] Storz G Altuvia S Wassarman KM An abundance of RNA regulators Annu Rev Biochem 2005 74 199 217 10.1146/annurev.biochem.74.082803.133136 15952886

[B10] Livny J Waldor MK Identification of small RNAs in diverse bacterial species Curr Opin Microbiol 2007 10 96 101 10.1016/j.mib.2007.03.005 17383222

[B11] Hüttenhofer A Vogel J Experimental approaches to identify non-coding RNAs Nucleic Acids Res 2006 34 635 646 10.1093/nar/gkj469 16436800 PMC1351373

[B12] Altuvia S Identification of bacterial small non-coding RNAs: experimental approaches Curr Opin Microbiol 2007 10 257 261 10.1016/j.mib.2007.05.003 17553733

[B13] Gottesman S Micros for microbes: non-coding regulatory RNAs in bacteria Trends Genet 2005 21 399 404 10.1016/j.tig.2005.05.008 15913835

[B14] Silvaggi JM Perkins JB Losick R Genes for small, noncoding RNAs under sporulation control in *Bacillus subtilis* J Bacteriol 2006 188 532 541 10.1128/JB.188.2.532-541.2006 16385044 PMC1347314

[B15] Landt SG Abeliuk E McGrath PT Lesley JA McAdams HH Shapiro L Small non-coding RNAs in *Caulobacter crescentus* Mol Microbiol 2008 68 600 614 10.1111/j.1365-2958.2008.06172.x 18373523 PMC7540941

[B16] Mandin P Repoila F Vergassola M Geissmann T Cossart P Identification of new noncoding RNAs in *Listeria monocytogenes *and prediction of mRNA targets Nucleic Acids Res 2007 35 962 974 10.1093/nar/gkl1096 17259222 PMC1807966

[B17] Arnvig KB Young DB Identification of small RNAs in *Mycobacterium tuberculosis* Mol Microbiol 2009 73 397 408 10.1111/j.1365-2958.2009.06777.x 19555452 PMC2764107

[B18] Livey J Brencic A Lory S Waldor MK Identification of 17 *Pseudomonas aeruginosa *sRNAs and prediction of sRNA-encoding genes in 10 diverse pathogens using the bioinformatic tool sRNAPredict2 Nucleic Acids Res 2006 34 3484 3493 10.1093/nar/gkl453 16870723 PMC1524904

[B19] Padalon-Brauch G Hershberg R Elgrably-Weiss M Baruch K Rosenshine I Margalit H Altuvia S Small RNAs encoded within genetic islands of *Salmonella typhimurium *show host-induced expression and role in virulence Nucleic Acids Res 2008 36 1913 2197 10.1093/nar/gkn050 18267966 PMC2330248

[B20] del Val C Rivas E Torres-Quesada O Toro N Jiménez-Zurdo JI Identification of differentially expressed small non-coding RNAs in the legume endosymbiont *Sinorhizobium meliloti *by comparative genomics Mol Microbiol 2007 66 1080 1091 10.1111/j.1365-2958.2007.05978.x 17971083 PMC2780559

[B21] Ulvé VM Sevin EW Chéron A Barloy-Hubler F Identification of chromosomal alpha-proteobacterial small RNAs by comparative genome analysis and detection in *Sinorhizobium meliloti *strain 1021 BMC Genomics 2007 19 467 10.1186/1471-2164-8-467 PMC2245857 18093320

[B22] Pichon C Felden B Small RNA genes expressed from *Staphylococcus aureus *genomic and pathogenicity islands with specific expression among pathogenic strains Proc Natl Acad Sci USA 2005 102 14249 14254 10.1073/pnas.0503838102 16183745 PMC1242290

[B23] Lenz DH Mok KC Lilley BN Kulkarni RV Wingreen NS Bassler BL The small RNA chaperone Hfq and multiple small RNAs control quorum sensing in *Vibrio harveyi *and *Vibrio cholerae* Cell 2004 118 69 82 10.1016/j.cell.2004.06.009 15242645

[B24] Zhang Y Zhang Z Ling L Shi B Chen R Conservation analysis of small RNA genes in *Escherichia coli* Bioinformatics 2004 20 599 603 10.1093/bioinformatics/btg457 15033865

[B25] Vogel J Sharma CM How to find small non-coding RNAs in bacteria Biol Chem 2005 386 1219 1238 10.1515/BC.2005.140 16336117

[B26] Hayward AC Swings JG, Civerolo EL The host of *Xanthomonas* Xanthomonas 1993 London: Chapman and Hall 51 54

[B27] He YQ Zhang L Jiang BL Zhang ZC Xu RQ Tang DJ Qin J Jiang W Zhang X Liao J Cao JR Zhang SS Wei ML Liang XX Lu GT Feng JX Chen B Cheng J Tang JL Comparative and functional genomics reveals genetic diversity and determinants of host specificity among reference strains and a large collection of Chinese isolates of the phytopathogen *Xanthomonas campestris *pv. *campestris* Genome Biol 2007 8 R218 10.1186/gb-2007-8-10-r218 17927820 PMC2246292

[B28] Kennedy JF Bradshaw IJ Production, properties and applications of xanthan Prog Ind Microbiol 1984 19 319 371

[B29] da Silva AC Ferro JA Reinach FC Farah CS Furlan LR Quaggio RB Monteiro-Vitorello CB Van Sluys MA Almeida NF Alves LM do Amaral AM Bertolini MC Camargo LE Camarotte G Cannavan F Cardozo J Chambergo F Ciapina LP Cicarelli RM Coutinho LL Cursino-Santos JR El-Dorry H Faria JB Ferreira AJ Ferreira RC Ferro MI Formighieri EF Franco MC Greggio CC Gruber A Katsuyama AM Kishi LT Leite RP Lemos EG Lemos MV Locali EC Machado MA Madeira AM Martinez-Rossi NM Martins EC Meidanis J Menck CF Miyaki CY Moon DH Moreira LM Novo MT Okura VK Oliveira MC Oliveira VR Pereira HA Rossi A Sena JA Silva C de Souza RF Spinola LA Takita MA Tamura RE Teixeira EC Tezza RI Trindade dos Santos M Truffi D Tsai SM White FF Setubal JC Kitajima JP Comparison of the genomes of two *Xanthomonas *pathogens with differing host specificities Nature 2002 417 459 463 10.1038/417459a 12024217

[B30] Qian W Jia Y Ren SX He YQ Feng JX Lu LF Sun Q Ying G Tang DJ Tang H Wu W Hao P Wang L Jiang BL Zeng S Gu WY Lu G Rong L Tian Y Yao Z Fu G Chen B Fang R Qiang B Chen Z Zhao GP Tang JL He C Comparative and functional genomic analyses of the pathogenicity of phytopathogen *Xanthomonas campestris *pv. *campestris* Genome Res 2005 15 757 767 10.1101/gr.3378705 15899963 PMC1142466

[B31] Mole BM Baltrus DA Dangl JL Grant SR Global virulence regulation networks in phytopathogenic bacteria Trends Microbiol 2007 15 363 371 10.1016/j.tim.2007.06.005 17627825

[B32] Dow M Diversification of the function of cell-to-cell signaling in regulation of virulence within plant pathogenic Xanthomonads Sci Signal 2008 1 pe23 10.1126/stke.121pe23 18506032

[B33] Vorhölter FJ Schneiker S Goesmann A Krause L Bekel T Kaiser O Linke B Patschkowski T Rückert C Schmid J Sidhu VK Sieber V Tauch A Watt SA Weisshaar B Becker A Niehaus K Pühler A The genome of *Xanthomonas campestris *pv. *campestris *B100 and its use for the reconstruction of metabolic pathways involved in xanthan biosynthesis J Biotechnol 2008 134 33 45 10.1016/j.jbiotec.2007.12.013 18304669

[B34] Daniels MJ Barber CE Turner PC Sawczyc MK Byrde RJW Fielding AH Cloning of genes involved in pathogenicity of *Xanthomonas campestris *pv. *campestris *using the broad-host-range cosmid pLAFR1 EMBO J 1984 3 3323 3328 16453595 10.1002/j.1460-2075.1984.tb02298.x PMC557857

[B35] Wengelnik K Marie C Russel M Bonas U Expression and localization of HrpA1, a protein of *Xanthomonas campestris *pv. *vesicatoria *essential for pathogenicity and induction of the hypersensitive reaction J Bacteriol 1996 178 1061 1069 8576039 10.1128/jb.178.4.1061-1069.1996 PMC177766

[B36] Ding Y Chan CY Lawrence CE Sfold web server for statistical folding and rational design of nucleic acids Nucleic Acids Res 2004 32 W135 141 10.1093/nar/gkh449 15215366 PMC441587

[B37] Cao Y Zhao YL Cha L Ying XM Wang LG Shao NS Li WJ sRNATarget: a web server for prediction of bacterial sRNA targets Bioinformation 2009 3 364 366 19707302 10.6026/97320630003364 PMC2720669

[B38] Altschul SF Gish W Mille W Myers EW Lipman DJ Basic local alignment search tool J Mol Biol 1990 215 403 410 2231712 10.1016/S0022-2836(05)80360-2

[B39] Yanisch-Perron C Vieira J Messing J Improved M13 phage cloning vectors and host strains: nucleotide sequences of the M13mp18 and pUC19 vectors Gene 1985 33 103 119 10.1016/0378-1119(85)90120-9 2985470

[B40] Miller JH Experiments in Molecular Genetics 1972 New York: Cold Spring Harbour Laboratory Press

[B41] Daniels MJ Barber CE Turner PC Cleary WG Sawczyc MK Isolation of mutants of *Xanthomonas campestris *pathovar *campestris *showing altered pathogenicity J Gen Microbiol 1984 130 2447 2455

[B42] Rochester DE Winer JA Shah DM The structure and expression of maize genes encoding the major heat-shock protein, Hsp70 EMBO J 1986 5 451 458 16453670 10.1002/j.1460-2075.1986.tb04233.x PMC1166785

[B43] Sambrook J Fritsch EF Maniatis T Molecular cloning: a laboratory manual 1989 2 Cold Spring Harbor, NY: Cold Spring Harbor Laboratory

