# Supplementary material for: Identification of four novel small non-coding RNAs from Xanthomonas campestris pathovar campestris
Source: BMC Genomics. 2010 May 20;11:316. doi: 10.1186/1471-2164-11-316 (PMC2996969; doi:10.1186/1471-2164-11-316)
Supplement: Additional file 1 — Figure S1. Schematic diagram showing the method used to clone Xcc sRNAs. [file 1471-2164-11-316-S1.DOC]

**Jiang et al., BMC Genomics. Additional Figure S1**

**Additional Figure S1. Schematic diagram showing the method used to clone *Xcc* sRNAs.** The TaKaRa small RNA cloning kit DRR065 (TaKaRa, Dalian, China) was used and the experimental steps were performed according to the manufacturer’s instructions.
