# Supplementary material for: Identification of four novel small non-coding RNAs from Xanthomonas campestris pathovar campestris
Source: BMC Genomics. 2010 May 20;11:316. doi: 10.1186/1471-2164-11-316 (PMC2996969; doi:10.1186/1471-2164-11-316)
Supplement: Additional file 5 — Table S8. Primers used in this study.doc [file 1471-2164-11-316-S5.DOC]

**Table S8.** Oligonucleotides, primers and RNA adaptors used in this work

| **Oligonucleotides or primers** | **Sequence (5’ to 3’)** | **source** |
| --- | --- | --- |
| **For cDNA library construction**  3’ Sse8387 I-Adaptor (RNA)  5’ Sse8387 I-Adaptor (RNA)  Sse8387-I specific reverse primer  (also used as RT-Primer)  Sse8387-I specific forward primer  **For cDNA clone screening**  M13 f/ M13 erv  **For sequencing**  M13 erv  **For northern blotting^Z^**  Probe-1(for sRNA-C1)  Probe-2 (for sRNA-C2)  Probe-3 for sRNA-C3)  Probe-4(for sRNA-C4)  Probe-5(for sRNA-C5)  Probe-6(for sRNA-C6)  Probe-7(for sRNA-C7)  **For** 5’**-Race mapping^Z^**  Abridged Anchor Primer (AAP)  AUAP  1GSP5-1(sRNA-*Xcc*1 specific)  1GSP5-2(sRNA-*Xcc*1 specific)  1GSP5-3(sRNA-*Xcc*1 specific)  2GSP5-1(sRNA-*Xcc*2 specific)  2GSP5-2(sRNA-*Xcc*2 specific)  2GSP5-3(sRNA-*Xcc*2 specific)  3GSP5-1(sRNA-*Xcc*3 specific)  3GSP5-2(sRNA-*Xcc*3 specific)  3GSP5-3(sRNA-*Xcc*3 specific)  4GSP5-1(sRNA-*Xcc*4 specific)  4GSP5-2(sRNA-*Xcc*4 specific)  4GSP5-3(sRNA-*Xcc*4 specific)  **For** 3’**-Race mapping^Z^**  3’**-** Adaptor (RNA)  3’Adaptor specific reverse primer  (also used as RT-Primer)  1GSP3-1(sRNA-*Xcc*1 specific)  1GSP3-2(sRNA-*Xcc*1 specific)  2GSP3-1(sRNA-*Xcc*2 specific)  2GSP3-1(sRNA-*Xcc*2 specific)  3GSP3-1(sRNA-*Xcc*3 specific)  3GSP3-2(sRNA-*Xcc*3 specific)  4GSP3-1(sRNA-*Xcc*4 specific)  4GSP3-2(sRNA-*Xcc*4 specific) | Biotin-CAUCGAUCCUGCAGGCUAGAGAC  AAAGAUCCUGCAGGUGCGUCA  GTCTCTAGCCTGCAGGATCGATG  AAAGATCCTGCAGGTGCGTCA  GTTTTCCCAGTCACGAC/AACAGCTATGACCATG  AACAGCTATGACCATG  Biotin-GGCGGCCGGGTGCTTGAACACGGTCGTAGGGCCGcccgcagTTTTCCCCTTTCGGGTAT  Biotin- TTGCATTCTCCGCCATGAC  Biotin- TCGATGCCGTTGCTGCCTTCCGGCCCT  Biotin- CCCCTGGCGATGACCTACTCTCGCATGGCTTGAGC  Biotin- CCAGGGGATGGGAGAGATCT  Biotin- TGGTCAAGCCGCACGGATCATTAG  Biotin-GGAGGTGATCCAGCCGCACCTTCCGATACGGCTACCTTGTTCCGACTCCACCCCAGT  GGCCACGCGTCGACTAGTACGGGIIGGGIIGGGIIG (I:deoxyinosine)  GGCCACGCGTCGACTAGTAC  GCGGCCGGGTGCTTG  TGCTTGAACACGGTCGTAGGGCG  AGGGCGCTCGCAGTTTTCCCCTTT  TTGCATTCTCCGCCATGA  CCGCCATGACGATGTGTGCGA  GGACGTTGTTAGGCCGTCGGGC  GGTGTCCCCGCCGGTT  TCCCCGCCGGTTGACCTCG  TGACCTCGGCGCCCGCATC  GCCCCTGGCGATGAC  GGCGATGACCTACTCTCGCATG  GACCTACTCTCGCATGGCTTGAG  Biotin-CAUCGAUCCUGCAGGCUAGAGAC  GTCTCTAGCCTGCAGGATCGATG  GGCCGGGTGGAAGGGCAGCTAT  GCAATACCCGAAAGGGGAAAACTGCG  GCGTTCTCTGCTGTGAACGACGGTGTG  CGGTGTGCAAAACATTCGCCTTGT  CCGCCAGGGCCGGAAGGCA  AGGCAGCAACGGTATCGATTGATG  CTCAAGCCATGCGAGAGTAGGTCAT  ATGCGAGAGTAGGTCATCGCCAGG | Takara, Dalian, China  Takara, Dalian, China  Takara, Dalian, China  Takara, Dalian, China  Sangon, Shanghai, China  Sangon, Shanghai, China  This work  This work  This work  This work  This work  This work  This work  Invitrogen, Carlsbad, USA  Invitrogen, Carlsbad, USA  This work  This work  This work  This work  This work  This work  This work  This work  This work  This work  This work  This work  Takara, Dalian, China  Takara, Dalian, China  This work  This work  This work  This work  This work  This work  This work  This work |

^z^ These primers were designed according to the genomic sequence of *X. campestris* pv. *campestris* strain 8004 [S1], and were synthesized and 5’-biotin labeled by Sangon Biological Engineering Technology & Services Co., Ltd (Shanghai, China).

S1. Qian W, Jia Y, Ren SX, He YQ, Feng JX, Lu LF, Sun Q, Ying G, Tang DJ, Tang H, Wu W, Hao P, Wang L, Jiang BL, Zeng S, Gu WY, Lu G, Rong L, Tian Y, Yao Z, Fu G, Chen B, Fang R, Qiang B, Chen Z, Zhao GP, Tang JL, He C: **Comparative and functional genomic analyses of the pathogenicity of phytopathogen *Xanthomonas campestris* pv. *campestris.*** *Genome Res* 2005, **15:**757-767.
